# Case Report: Esophago-pleural fistula found by the squamous cells from pleural effusion

**DOI:** 10.3389/fonc.2025.1647915

**Published:** 2025-10-08

**Authors:** Shu Li, Zesen Han, Xue Zhang, Qihui Ge, Yingying Zhao, Shaojuan Lv, Min Li, Yongchao Zhu

**Affiliations:** ^1^ Department of Clinical Laboratory, Hua County People’s Hospital, Anyang, Henan, China; ^2^ Department of Pathology, Hua County People’s Hospital, Anyang, Henan, China; ^3^ Department of Oncology II, Hua County People’s Hospital, Anyang, Henan, China; ^4^ Department of Ultrasonography, Hua County People’s Hospital, Anyang, Henan, China; ^5^ Department of Computerized Tomography, Hua County People’s Hospital, Anyang, Henan, China

**Keywords:** esophago-pleural fistula, squamous cells, pleural effusion, esophageal cancer, hydropneumothorax

## Abstract

This report describes a case of esophago-pleural fistula (EPF) in an history of esophageal cancer (EC) patient, initially missed on CT but identified via squamous cells in pleural effusion. EPF is rare and challenging to diagnose due to subtle symptoms. The presence of squamous cells in pleural effusion, typically linked to lung cancer, initially confused the diagnosis given the patient’s EC history. Methylene blue ingestion and clinical evaluation confirmed EPF. This case emphasizes the value of pleural fluid analysis in malignancy patients and the need for vigilance in EC patients, especially with radiation-induced esophagitis.

## Introduction

Esophageal cancer (EC) is the seventh leading cause of cancer-related mortality worldwide (1). Esophageal fistula may occur as a complication associated with this malignancy (2). Esophago-pleural fistula (EPF) represents a rare subtype of esophago-respiratory fistulas (ERF) (3). EPF can arise due to various risk factors, which are often identified through computed tomography (CT), alongside symptoms such as fever, tachycardiac, and tachypneic (4). However, early identification of EPF remains challenging, frequently resulting in adverse outcomes.

Traditionally, pleural effusion has been most indicative of positive tumor diagnoses for adenocarcinoma and less so for mesothelioma, squamous cell carcinoma, lymphoma, and sarcoma (5). The prognosis for patients with squamous cell carcinoma tends to be poorer (6). Consequently, normal squamous cells can rarely be detected in pleural effusions from lung or esophageal cancer cases, their presence in a patient with a history of EC can lead to diagnostic confusion.

This study presents a case of an esophago-pleural fistula that was overlooked on CT imaging due to excessive hydrothorax; however, the detection of squamous cells within the pleural effusion suggested the existence of EPF.

## Case presentation

A 67-year-old male patient presented to the hospital with fever reaching 38.5°C and right-sided chest pain lasting one day. His diagnosis of esophageal squamous cell carcinoma was confirmed by pathological microscopy and was clinically staged as cT3N1M0 (stage IIIB). From July 11 to August 23, 2023, he underwent radiotherapy totaling 63 Gy over 35 sessions. At presentation, he exhibited radiation-induced esophagitis but showed improvement following symptomatic treatment. Concurrently, the patient received four cycles of chemotherapy consist of paclitaxel albumin-bound formulation at 300mg on day one, carboplatin at 300mg on day two, along with camrelizumab therapy. The effect evaluation indicted stable disease (SD). On January 10, 2024, the patient began monotherapy with camrelizumab (200 mg) for antitumor treatment. After one cycle, a decrease in thyroid-stimulating hormone level was observed. Immunotherapy was subsequently discontinued, and intravenous methylprednisolone (40 mg) was initiated as hormonal therapy. On July 5, 2024, a follow-up examination using a 64-slice CT scan revealed an estimated partial response (PR). Following this assessment, the patient underwent three cycles of Anlotinib targeted therapy.

**Figure 1:**
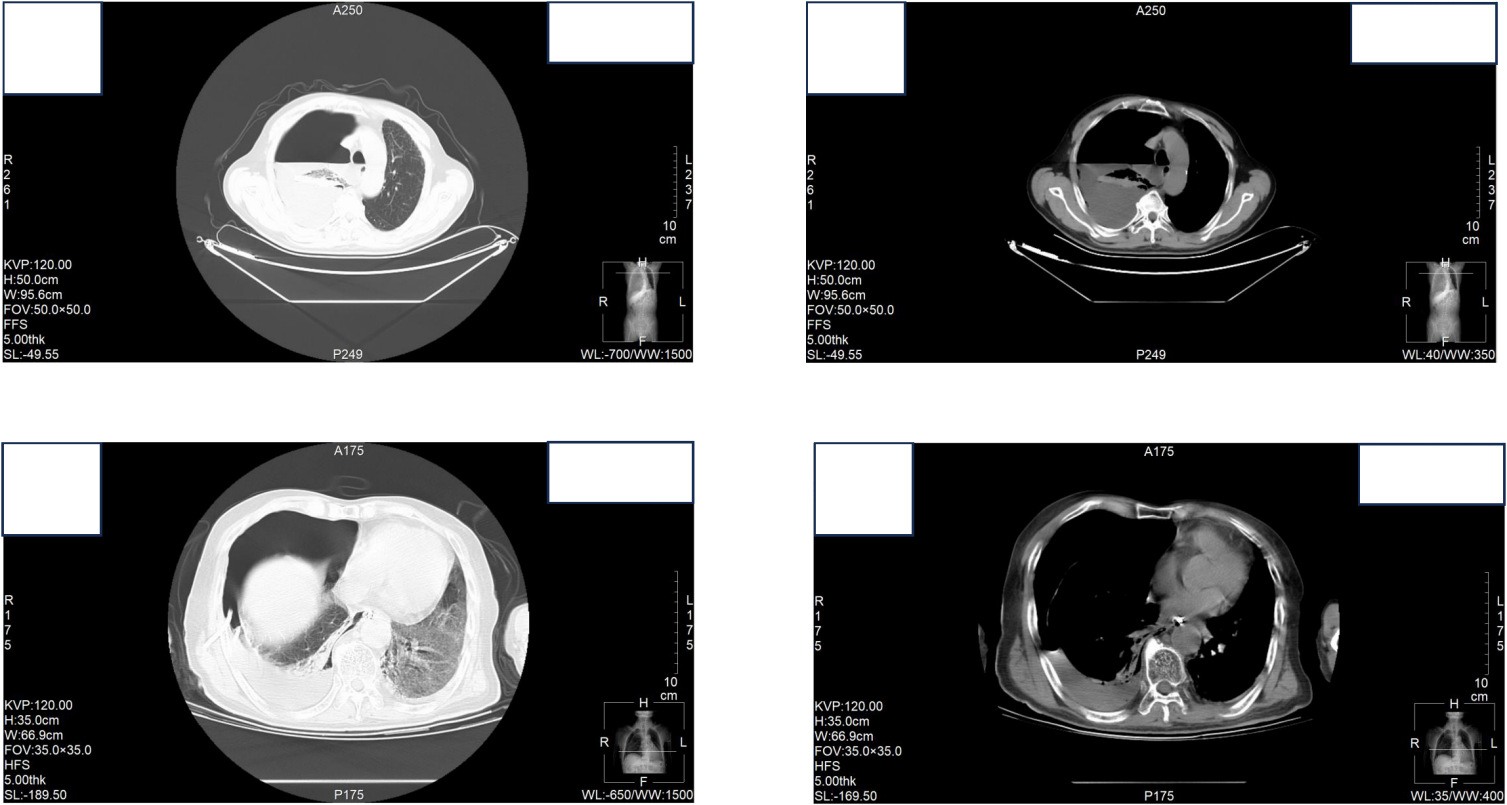
Contrast-enhanced CT scan of the chest demonstrated pleural effusion and hydropneumothorax in the right thoracic cavity.

**Figure 2:**
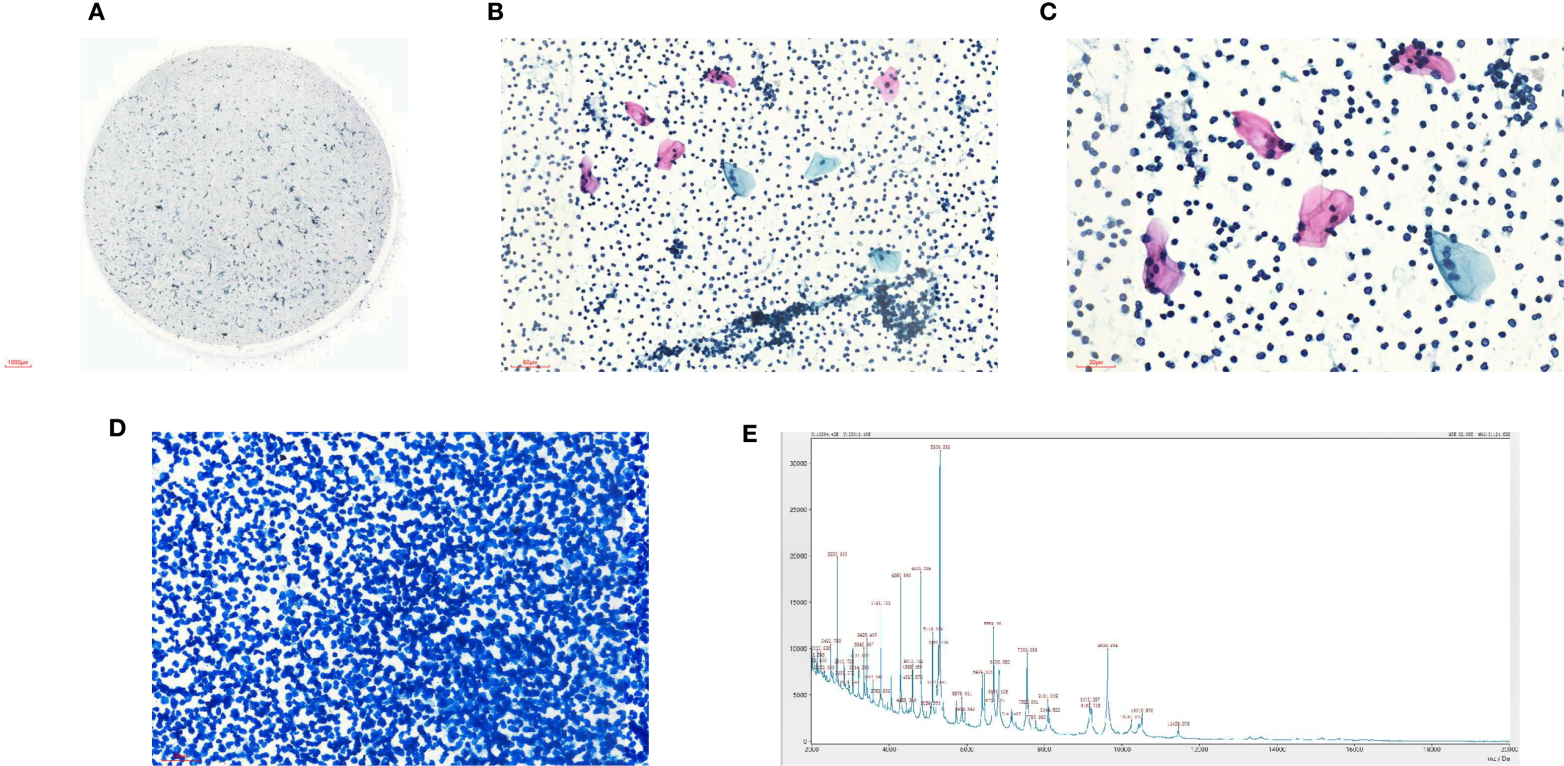
**(A)** Liquid based cytology of pleural effusion (40X); **(B)** Liquid based cytology of pleural effusion (100X); **(C)** Liquid based cytology of pleural effusion (200X); **(D)** Acid-fast staining (100X); **(E)** Microbial mass spectrometry analysis.

**Figure 3:**
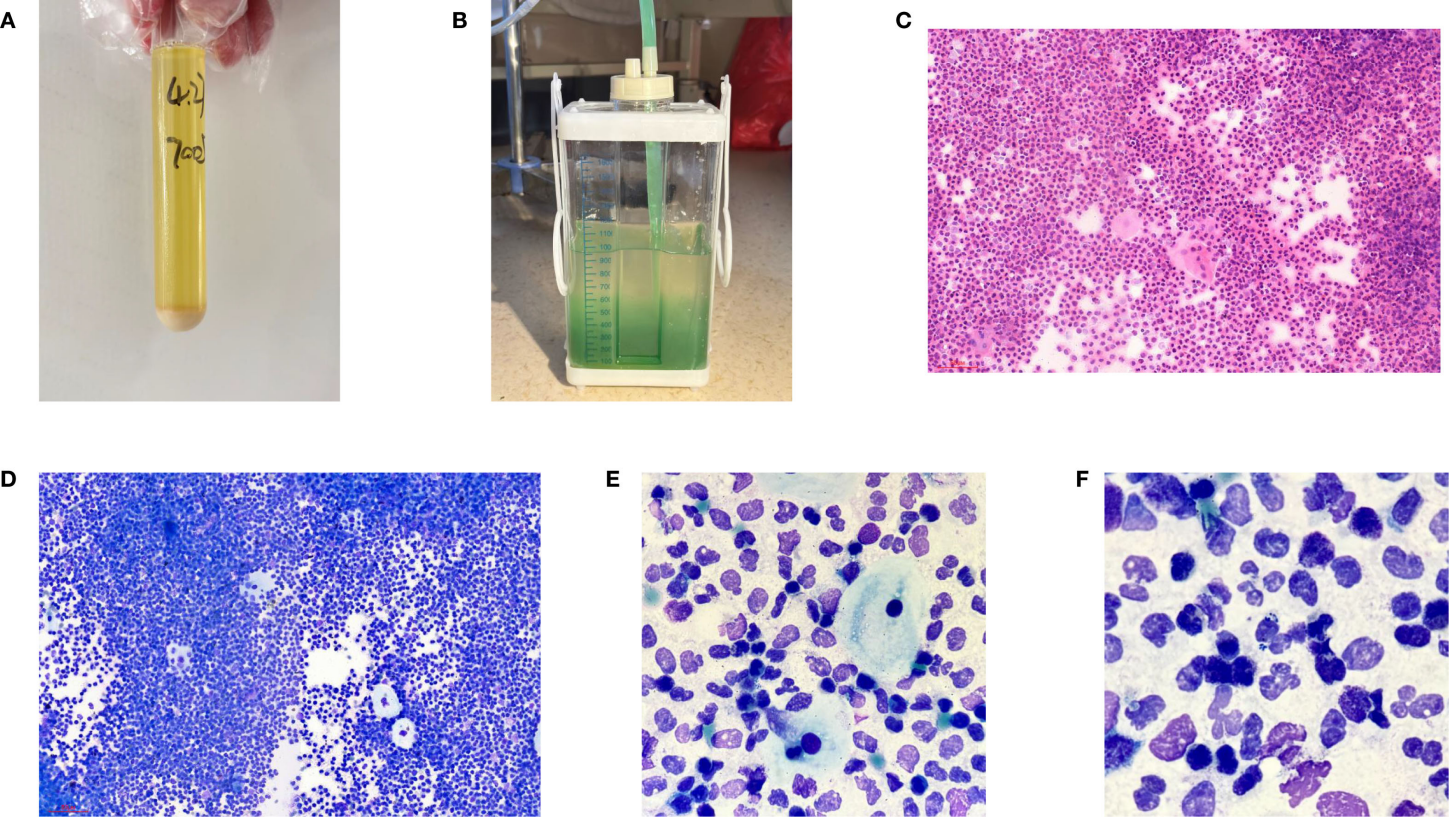
**(A)** General manifestations of pleural effusion; **(B)** Performance of the drainage bottle after Methylene blue ingestion. **(C)** Hematein-Eosin stain of pleural effusion(X200); **(D)** Ray-giemsa stain of pleural effusion(X200); **(E, F)** Ray-giemsa stain of pleural effusion(X1000) showed the squamous cells, neutrophils granulocyte and bacteria.

At the time of hospitalization, her vital signs were stable (temperature 36.5 °C, pulse rate 67 bpm, blood pressure 133/68 mm Hg, respiratory rate 18 breaths/min). The physical examination of the respiratory system revealed that breath sounds in the right lung were slightly diminished. No other positive clinical signs were noted. A contrast-enhanced CT scan of the chest demonstrated pleural effusion and hydropneumothorax in the right thoracic cavity (**Figure 1**). There was no evidence of metastatic lesions, lung parenchymal lesions or regional adenopathy on the CT scan. Therefore, it is possible that EPF may be obscured. Consequently, thoracic drainage was performed to collect hydrothorax for laboratory and pathologic inspection. The laboratory physician identified normal squamous cells within the hydrothorax and communicated these findings to the clinical team. The indictors of Interleukin-6, tumor markers, routine and biochemical test of pleural effusion indicted the inflammation (**Table 1**). The leukocyte count was recorded at 14.26*10^9/L with neutrophils constituting approximately 90.80% of total white blood cells. The Rivalta test for the pleural effusion yield a positive result. The nucleated cell count number was at 17343*10^6/L. The chlorine level was recorded at 105.1 mmol/L, while the glucose level was found to be 3.45 mmol/L. The total protein concentration was less than 20.00 g/L (**Table 1**). Simultaneously, acid-fast staining and microbial mass spectrometry analysis were conducted by the laboratory personnel (**Figure 2**). The appearance of fluid was yellow and turbid; typically, it is faint yellow and transparent (**Figure 3A**). Negative results from acid-fast staining effectively ruled out Mycobacterium tuberculosis infection while microbial mass spectrometry confirmed an infection with Staphylococcus epidermidis. Pathological exfoliated cytology did not reveal any tumor cells nor evidence of cancer recurrence.

**Table 1:** Routine and biochemical test and tumor markers of pleural effusion.

Items	Measured values	Reference values
Interleukin-6 (IL-6)	606.631 pg/ml	0--6.6
Alpha Fetoprotein (AFP)	2.534 ng/ml	0--10
Carcino-embryonic Antigen (CEA)	0.649 ng/ml	0--5
Carbohydrate Antigen CA24-2	3.701 U/ml	0--25
Carbohydrate Antigen CA-50	2.737 U/ml	0--25
Squamous Cell Carcinoma Antigen (SCCA)	0.287 ng/ml	0--1.5
Carbohydrate Antigen CA19-9	<2.000 U/ml	0--35
Carbohydrate Antigen CA724	3.601 U/ml	0--10
Colour	Yellow	Faint Yellow
Transparency	Turbidity	Transparent
Rivalta	Positive (+++)	Negative
Nucleated Cells Counts	17343*10^6/L	0-100
Total Number of Cells Observed	100	
Percentage of Lymphocytes	21%	
Percentage of Phagocytes	71%	
Percentage of Eosinophilic Granulocyte	8%	
Percentage of Basophilic Granulocyte	None	
Percentage of Mesothelial Cells	None	
Percentage of Abnormal Cells	None	
Chlorine	105.1 mmol/L	99-110 mmol/L
Glucose	3.45 mmol/L	2.2--3.9 mmol/L
Total Protein	<20.00 g/L	65--85 g/L

In light of uncertainties regarding the sources of squamous cells (**Figure 3**) detected in hydrothorax samples, collaboration ensued between laboratory physicians and clinical doctors alongside CT technicians who administered methylene blue orally to assess potential connections to esophageal pathology. The subsequent observation that chest drainage fluid turned blue corroborated the presence of an esophago-pleural fistula (**Figure 3**). Management included nasogastric intubation, active drainage, and a course of anti-infective therapy consisting of vancomycin (1 g administered every 12 hours for 8 days), in addition to nutritional support and other adjunctive interventions. Clinical symptoms improved significantly following these treatments. Due to his poor physical health, further intervention measures could not be undertaken for this patient. Additionally, a fistula closure had not been performed prior to discharge from the hospital as requested by his family members. Unfortunately, he passed away on May 12, 2025.

## Discussion

The present case underscores the diagnostic challenges and management considerations associated with esophago-pleural fistula (EPF), a rare yet potentially life-threatening complication of esophageal cancer (EC). This case emphasizes the importance of a multidisciplinary approach in both diagnosing and treating such complex conditions.

In this patient, the diagnosis of EPF was initially overlooked on CT imaging, underscoring the limitations of imaging modalities in detecting subtle fistulas. Despite the patient’s history of EC and ongoing treatment, the fistula remained undetected until pleural effusion analysis revealed the presence of squamous cells. This finding accentuates the significance of pleural fluid analysis in identifying underlying pathologies, particularly among patients with a malignancy history. The detection of squamous cells in the pleural effusion, typically associated with lung cancer, initially caused confusion given the patient’s background with EC. However, subsequent ingestion of methylene blue, along with clinical correlation, ultimately confirmed the diagnosis of EPF.

Management strategies for EPF necessitate a comprehensive approach that includes addressing the underlying malignancy, controlling infection, and providing nutritional support (7). Furthermore, employing a tailored multimodal strategy (such as stenting combined with over-the-scope clips) may prove to be safer and more effective than relying on a single modality alone while potentially avoiding surgical intervention (8). In this instance, thoracic drainage was performed alongside antibiotic therapy for infection control and nutritional support; these interventions contributed significantly to an improvement in clinical symptoms. Additionally, nasogastric tube catheterization facilitated active drainage and assisted in managing the fistula effectively. These measures highlight the importance of timely intervention and collaborative efforts from a multidisciplinary team when managing EPF.

Furthermore, this case underscores the necessity for heightened vigilance in patients with a history of EC. The patient’s prior experience with radiation-induced esophagitis may elevate the risk of fistula formation. Additionally, T4 stage and baseline esophageal stenosis independently contribute to an increased the risk of developing esophageal fistula (2). Benign EPF is rare and can be result from trauma or infection with tuberculosis being the most common infectious cause. In this instance, specific infections were ruled out due to negative result.

Moreover, there are multiple potential causes for esophago-pleural fistula, including spontaneous occurrence (9), pill-induced esophagitis resulting from calcium supplements (10), among others. If the CT fails to identify the diagnosis, squamous cells present in pleural effusion may serve as an alert for clinician regarding the doctors. Last but not least, the earlier EPF is detected, the better the patients’ prognosis will be.

Regarding treatment options, nasogastric tube catheterization for feeding has shown potential to improve prognosis effectively (11).

Another factor that warrants consideration is the side effects associated with targeted therapies when combined with radiotherapy. Several critical incidents have been reported, however, information remains limited (12). Furthermore, data concerning safety and toxicity in elderly patients are scarce (13). Therefore, further research is essential to optimize treatment strategies.

## Conclusion

This case of EPF in a patient with EC highlights the diagnostic and therapeutic challenges posed by this rare complication. It emphasizes the significance of pleural fluid analysis in diagnosing underlying pathologies, particularly in individuals with a malignancy history. The integrated use of squamous epithelial cells found in pleural effusion alongside methylene blue imaging and PET/CT can effectively diagnose mediastinal leakage from the esophageal. This approach provides a foundation for early intervention regarding complications following radiotherapy combined with targeted therapy. The presence of squamous cells within pleural effusion could serve as an important indicator for EPF.

## Data Availability

The original contributions presented in the study are included in the article/supplementary material. Further inquiries can be directed to the corresponding authors.
